# Host metabolic shift during systemic *Salmonella* infection revealed by comparative proteomics

**DOI:** 10.1080/22221751.2021.1974316

**Published:** 2021-09-17

**Authors:** Yuanyuan Wang, Chunmei Wu, Jiacong Gao, Xudong Du, Xiangyun Chen, Mei Zhang

**Affiliations:** a TEDA School of Biological Sciences and Biotechnology, Nankai University, Tianjin, People’s Republic of China; b School of Chinese Materia Medica, Beijing University of Chinese Medicine, Beijing, People’s Republic of China

**Keywords:** *Salmonella* Typhimurium, mouse infection model, systemic infection, SPI-2, comparative proteomics

## Abstract

*Salmonella enterica* serovar Typhimurium (*S.* Typhimurium) is a food-borne bacterium that causes acute gastroenteritis in humans and typhoid fever in mice. *Salmonella* pathogenicity island II (SPI-2) is an important virulence gene cluster responsible for *Salmonella* survival and replication within host cells, leading to systemic infection. Previous studies have suggested that SPI-2 function to modulate host vesicle trafficking and immune response to promote systemic infection. However, the molecular mechanism and the host responses triggered by SPI-2 remain largely unknown. To assess the roles of SPI-2, we used a differential proteomic approach to analyse host proteins levels during systemic infections in mice. Our results showed that infection by WT *S.* Typhimurium triggered the reprogramming of host cell metabolism and inflammatory response. *Salmonella* systemic infection induces an up-regulation of glycolytic process and a repression of the tricarboxylic acid (TCA) cycle. WT-infected tissues prefer to produce adenosine 5′-triphosphate (ATP) through aerobic glycolysis rather than relying on oxidative phosphorylation to generate energy. Moreover, our data also revealed that infected macrophages may undergo both M1 and M2 polarization. In addition, our results further suggest that SPI-2 is involved in altering actin cytoskeleton to facilitate the *Salmonella*-containing vacuole (SCV) biogenesis and perhaps even the release of bacteria later in the infection process. Results from our study provide valuable insights into the roles of SPI-2 during systemic *Salmonella* infection and will guide future studies to dissect the molecular mechanisms of how SPI-2 functions *in vivo*.

## 1. Introduction

*Salmonella enterica* serovar Typhimurium (*S.* Typhimurium), a facultative gram-negative bacterium, is one of the leading causes of bacterial food-borne infections. *S.* Typhimurium infections cause gastroenteritis (self-limiting food poisoning) in human and the systemic typhoid-like infection in mice [1]. Human typhoid fever is caused by *Salmonella* Typhi with 21 million new cases and 200,000 deaths worldwide each year [2]. A streptomycin-pretreated mouse model of *S.* Typhimurium infection was established to study gastroenteritis and typhoid fever in mice and this is now widely used [3]. After ingestion of contaminated water or food, *S.* Typhimurium can overcome the acidic pH of the stomach and succeeds in reaching the small intestine, where it adheres to and invade the small intestinal epithelial cells [4]. Once *S.* Typhimurium crosses the intestinal epithelium, it may spread to the mesenteric lymph nodes and reach the liver and spleen, leading to more severe systemic infection. *Salmonella* has evolved complex mechanisms to enter and colonize host cells [5]. These virulence properties of *Salmonella* are mainly derived from *Salmonella* pathogenicity islands (SPIs), virulence pSLT plasmids, adhesins, and flagella [6–9]. In mice, *S.* Typhimurium invasion may occur through transcytosis mediated by microfolded cells (M cells) that overlying Peyer's Patches [10].

Once inside the host cell, *S.* Typhimurium can survive and replicate in a unique membrane-bound compartment called the *Salmonella*-containing vacuole (SCV). *Salmonella* Pathogenicity Island I (SPI-1) plays an essential role in *Salmonella* invasion by inducing actin cytoskeletal rearrangements, and may also be involved in the early stages during SCV maturation [11–13]. After crossing the Peyer's Patches, *Salmonella* reach the submucosa and may be endocytosed by phagocytic cells (e.g. macrophages) and reside within the SCVs inside the phagocytic cells. The infected phagocytes may enter the bloodstream and facilitate the transmission to other tissues/organs such as the liver, spleen and bone marrow [14]. *Salmonella* pathogenicity island II (SPI-2) is primarily responsible for the biogenesis of SCVs, thus contributing to the intracellular survival and replication of *Salmonella*, promoting systemic infections [15,16]. Bacterial multiplication in the liver and spleen triggers the formation of acute abscesses composed mainly of polymorphonuclear leukocytes. These lesions may ultimately lead to granulomata [17]. Most live *Salmonella* are situated inside the macrophages within these lesions during the late stages of infection, and few extracellular bacteria can be found [18,19].

During systemic infection, SPI-2 encoded virulence genes enable *S.* Typhimurium to survive and to replicate inside macrophages. Many of these virulence genes also elicit host immune responses and even cell death. Therefore, it is pivotal to study the nature of the host responses during systemic infection in order to understand how *Salmonella* overcome and promote its replication in the hostile macrophages. Proteomic analysis is a powerful approach to analyse complex biological samples and has been successfully used to study host responses during bacterial infections. For instance, Mijke *et al.* identified host proteins associated with intracellular *Salmonella* replication using a quantitative Stable Isotope Labeling by Amino acids in Cell culture (SILAC) approach [20]. Stephanie *et al.* described a novel proteome-based approach to enrich and characterize *Salmonella*-modified host cell membranes, and demonstrated the hitherto unrecognized complexity in the formation of the *Salmonella* host locus [21]. Liu’s group analysed epithelial cells during *Salmonella* infection and found that host glucose transporter proteins and glycolytic enzymes were significantly induced [22]. Joel *et al.* utilized Click chemistry with Pulsed SILAC to quantify host proteome profiles of *Salmonella*-infected macrophages. Aberrant trafficking of lysosomal proteases to the extracellular space and the nucleus were also detected [23].

**Figure 1.**
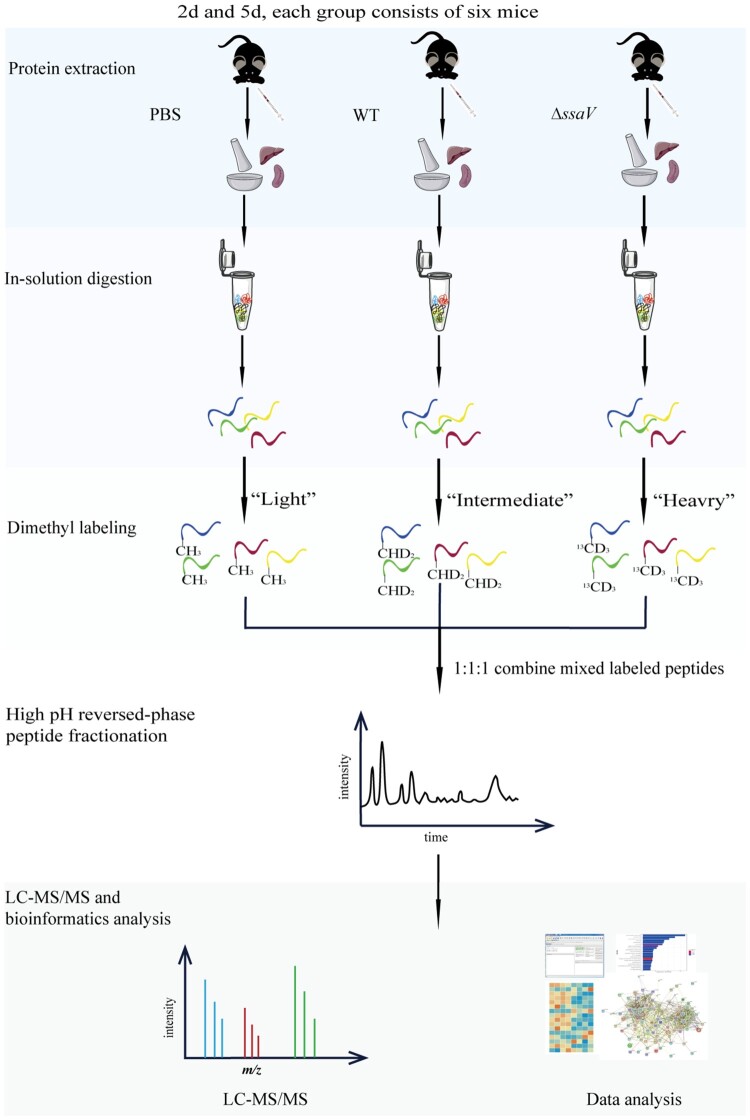
Workflow for comparative proteomics analysis based on stable isotope dimethyl-labelling. Streptomycin-pretreated mice were infected with WT *S.* Typhimurium SL1344 or Δ*ssaV* MT at 2 and 5 dpi. PBS-treated mice under the same conditions were used as controls. Protein samples from mouse liver and spleen in PBS, WT and *ΔssaV*-infected groups were extracted, digested, labelled, and mixed in a 1:1:1 ratio for LC-MS/MS analysis and subsequent data analysis.

Despite the progress made in elucidating the functions of many SPI-2 effectors and revealing the mechanism of *S.* Typhimurium infection, host responses triggered and the comprehensive roles of SPI-2 during systemic *S.* Typhimurium infection remain poorly understood. We employed the classical stable isotope dimethyl-labelling approach to study comparative host protein profiles during systemic *Salmonella* infections (Figure 1). The mouse infection model was used to compare the host response resulting from systemic infection of *S.* Typhimurium WT SL1344 with that of the Δ*ssaV* mutant, as deletion of the *ssaV* gene causes defection in SP-2 secretion [24]. A series of differentially expressed proteins (DEPs) have been identified, and the biological pathways in which they may be involved are analysed. Our comprehensive proteomic survey reveals the roles of SPI-2 and host adaptation during *S.* Typhimurium systemic infection and provides valuable clues for future studies on the biology of *Salmonella*-host interactions.

## 2. Materials and methods

The critical methods are briefly given in the main text, including peptide labelling methods and proteomic data processing and bioinformatics analysis. Due to the journal's word count limit for the main text, the detailed descriptions of other experiments in the “Materials and Methods” section are provided in the Supporting Information, including: bacterial strains and culture conditions; animal infection protocols; analysis of *S.* Typhimurium bacterial loads in tissues; histopathology evaluation; protein sample preparation; high-pH reversed-phase peptide fractionation; nanoflow LC-MS/MS analysis; proteomic data processing and bioinformatics analysis; and Western blot (WB).

### 2.1. Peptide stable isotope dimethyl-labelling

Stable isotope dimethyl-labelling of peptides was carried out according to the reported protocol [25]. PBS-treated, WT-infected and Δ*ssaV* MT-infected groups were “light”, “intermediate” and “heavy” labelled, respectively. Reverse-labelling has been investigated. When analysing samples at different time points, the light, intermedium and heavy labelled samples were mixed at 1:1:1 ratio.

### 2.2. Proteomic data processing and bioinformatics analysis

Raw files were processed by Proteome Discoverer 2.2 and searched against the proteome database of C57/BL6 mouse downloaded from Uniprot (http://www.uniprot.org/) according to the required conditions. Proteins with a fold-change > 1.5 or < 0.67 (*P* < 0.05) were considered as DEPs. The metascape database (https://metascape.org) was used to perform Gene Ontology (GO) annotation [26] and Kyoto Encyclopedia of Genes and Genomes (KEGG) pathway analysis [27].

## 3. Results

### 3.1. Bacterial load during systemic *S.* Typhimurium infection in mice

**Figure 2.**
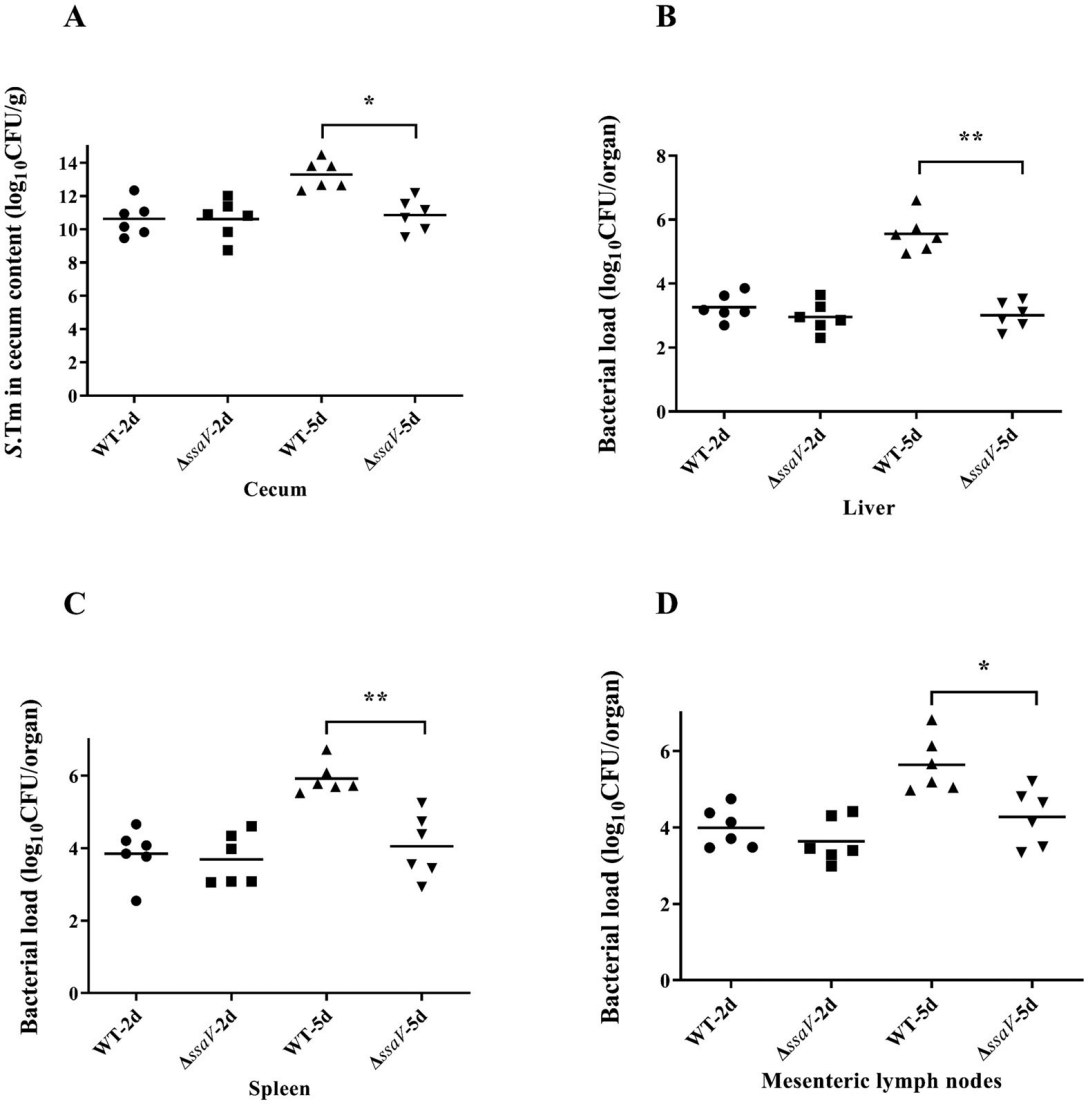
Bacterial loads in the organs of WT *S.* Typhimurium or Δ*ssaV* MT-infected mice were counted at 2 and 5 dpi: cecum contents (**A**), liver (**B**), spleen (**C**) and mesenteric lymph nodes (**D**). *, *P* < 0.05; **, *P* < 0.01, Mann-Whitney U-test.

To evaluate the host response during systemic *Salmonella* infection and the role of SPI-2 in the infection process, we used the Streptomycin-pretreated mouse model as described previously [3]. We first determined the appropriate time points that could represent the different stages of systemic infection by enumerating bacterial loads in various tissues coupled with histopathology evaluations. Streptomycin-pretreated C57BL/6 mice were infected with either the SL1344 WT or *ΔssaV* MT *Salmonella* strains. Bacterial loads in the liver, spleen, mesenteric lymph nodes, and cecal contents were enumerated at 2 days postinfection (dpi, early) or 5 dpi (late). No significant difference in bacterial loads were found at 2 dpi between the WT- and MT-infected groups in all the four tissues tested (Figure 2(A–D)). However, the bacterial loads in the liver, spleen, mesenteric lymph nodes and cecal contents from mice infected by the Δ*ssaV* MT strain was significantly lower than that of the WT strain at 5 dpi (Figure 2(A–D)). This is consistent with the previous report that intestinal bacterial loads and pathological changes were dramatically attenuated in mice infected with the SPI-2 mutant (Δ*ssaV*) compared to WT bacteria at 5 dpi [15].

### 3.2. Histopathological changes during *S.* Typhimurium systemic infection in mice

**Figure 3.**
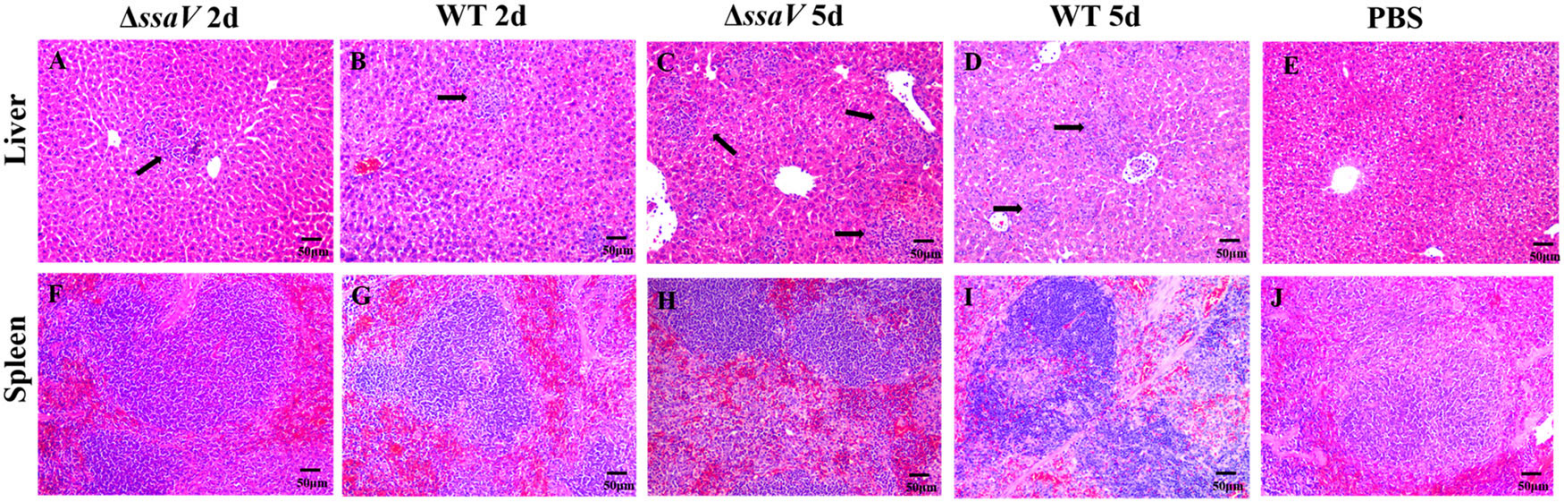
Hematoxylin and Eosin (H&E) stained histopathological sections of liver and spleen of WT *S.* Typhimurium or Δ*ssaV* MT-infected mice at 2 and 5 dpi (HE, ×100; Bar: 50 μm). Black arrows indicated infiltration of inflammatory cells.

Bacterial loads in *Salmonella* infected mice often have accompanying histopathological changes in tissues. We examined the four different organs in WT-infected mice and Δ*ssaV* MT-infected mice for histopathological changes. Mice were infected as described above and tissue samples (2 and 5 dpi) were fixed, embedded and stained with Hematoxylin and Eosin (H&E). Liver sections of mice infected with the WT or Δ*ssaV* MT showed very mild infiltration of inflammatory cells and some granuloma formation at 2 dpi (Figure 3(A,B)). At 5 dpi, severer hepatic necrosis were observed in liver section of WT-infected mice while focal necrosis infiltrated by neutrophils around multiple granulomas were observed from liver section of the Δ*ssaV* MT-infected mice (Figure 3(C,D)). At 2 dpi, spleens from WT-infected mice have obvious pathological changes while no noticeable changes were observed in spleen from MT-infected mice (Figure 3(F,G)). At 5 dpi, spleens from mice infected with both MT and WT strains showed enlarged splenic nodules, increased macrophages and splenic follicular necrosis in the white pulp area (Figure 3(H,I)). No significant changes were found in mesenteric lymph nodes of mice infected by either the WT or the MT at 2 dpi and 5 dpi. In addition, we also investigated the cecal samples. The histopathological changes of mice cecum were more pronounced than that in the liver and spleen after infection (Figure S1). These results demonstrate that WT-infected mice displayed more severe inflammatory response compared to that of Δ*ssaV*-MT infected mice, especially at 5 dpi.

### 3.3. Proteomic profiling of mice liver and spleen in response to *S.* Typhimurium systemic infection

To understand host responses to *S.* Typhimurium systemic infection, we used a stable isotope dimethyl-labelling quantitative proteomic approach to analyse protein levels in the liver and spleen of infected mice (Figure 1). Streptomycin-pretreated mice were infected with WT *S.* Typhimurium SL1344 or the SPI-2 defective mutant (Δ*ssaV* MT) as described above. Liver and spleen samples were taken at 2 dpi or 5 dpi.

**Figure 4.**
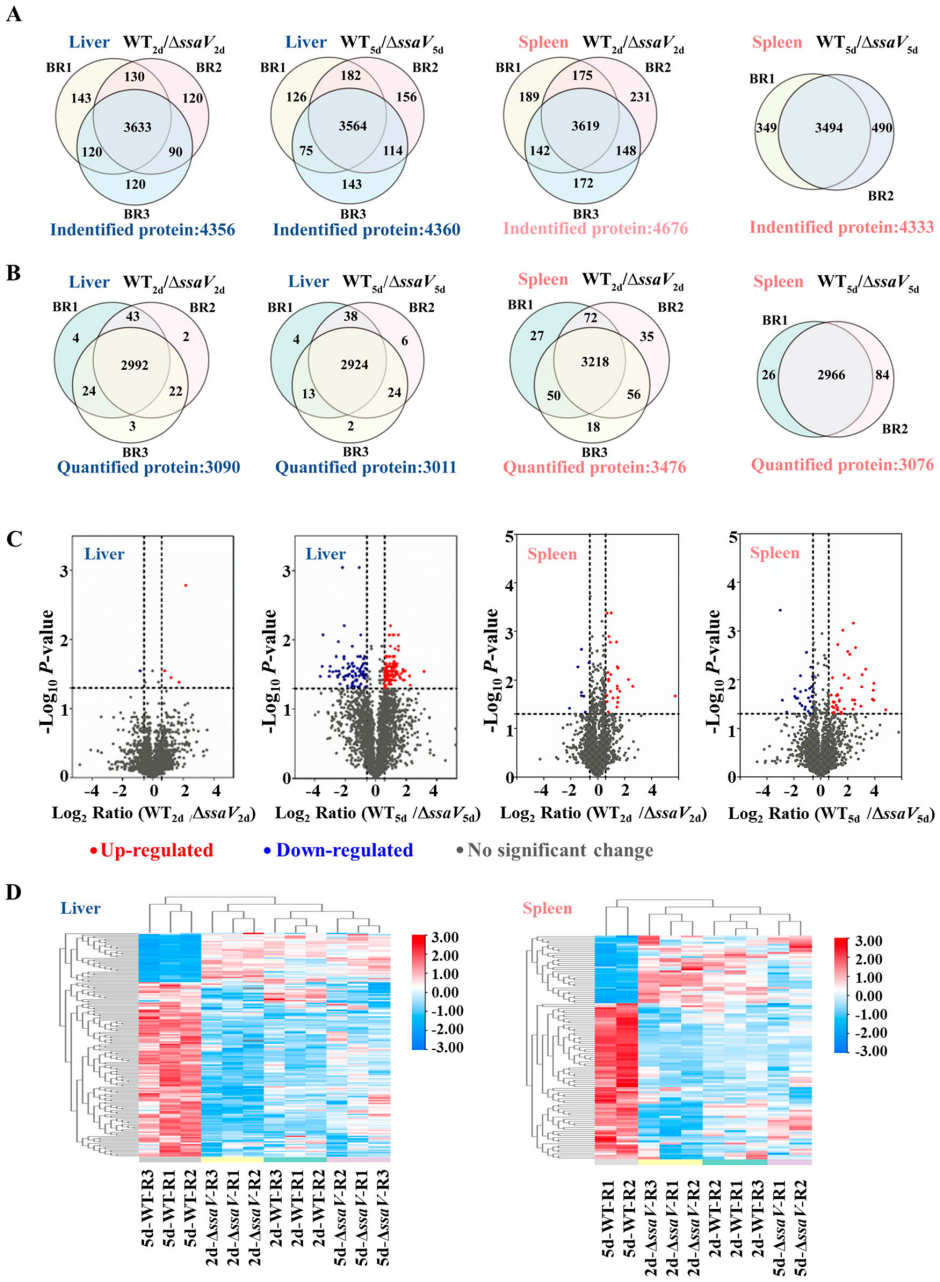
Protein profiles in the liver and spleen of *S.* Typhimurium-infected mice and alterations across early and late stages of systemic infection. “WT_xd_/Δ*ssaV*_xd_” refers to the mixture of WT-infected samples and Δ*ssaV* MT-infected samples at a 1:1 ratio at x days post-infection. Each circle represents the number of proteins identified or quantified in the mixed samples in one biological replicate. (**A**), Venn diagrams showing the number of proteins identified in each group of samples. (**B**), Venn diagrams showing the number of proteins that were quantified in each group of samples. (**C**), Volcano plots showing pairwise comparisons of protein expression levels between WT and Δ*ssaV* groups in mice liver or spleen at different stages of systemic infection. Points above horizontal dashed lines represent significantly altered proteins (two-sided t test, *P* < 0.05, *P* values were adjusted by Benjamini–Hochberg correction for multiple comparisons). Significantly up-regulated proteins are shown in red (protein fold change > 1.5) and down-regulated ones are shown in blue (protein fold change < 0.67). (**D**), Heatmap analysis of the identified proteins from each condition. Rows in heatmap denotes quantified proteins, and column denotes sample. Red colour denotes highly expressed protein, whereas blue denotes lowly expressed protein.

Proteins in each experimental condition (liver/spleen; WT/MT; 2 dpi/5 dpi) were quantitatively determined by nanoflow LC-MS/MS, and data were analysed as described above. In total, we identified 4356 and 4360 proteins from liver “WT_2d_/Δ*ssaV*_2d_” and “WT_5d_/Δ*ssaV*_5d_” samples in three biological replicates (BRs); 4676 and 4333 proteins were identified from spleen “WT_2d_/Δ*ssaV*_2d_” and “WT_5d_/Δ*ssaV*_5d_” samples, respectively (Figure 4(A)). A total of 3090 and 3011 proteins were quantified in the liver “WT_2d_/Δ*ssaV*_2d_” and “WT_5d_/Δ*ssaV*_5d_” samples. Likewise, 3476 and 3076 proteins were captured from the spleen “WT_2d_/Δ*ssaV*_2d_” and “WT_5d_/Δ*ssaV*_5d_” samples (Figure 4(B)). The volcano plots of WT vs. Δ*ssaV* MT groups were constructed to represent the quantitative data of protein levels under various experimental conditions (Figure 4(C)). Proteins with significantly (*P* < 0.05) different abundances are shown as dashed horizontal lines. Changes in protein levels of less than 0.67-fold or greater than 1.5-fold are shown to the left or right of the vertical dashed line respectively. Hierarchical clustering of protein levels in different tissues are plotted in Figure 4(D). A few DEPs were found between WT- and MT-infected groups at 2 dpi in both liver and spleen samples. However, at 5 dpi, WT *S.* Typhimurium induced more extensive protein changes than that of the SPI-2 deficient MT strain. The reproducibility of experiments are demonstrated by the low relative standard deviation (RSD) (Figure S2 in Supporting Information) and by the hierarchical clustering analysis in Figure 4(D).

### 3.4. Gene Ontology and KEGG pathway analysis of differentially expressed proteins

**Figure 5.**
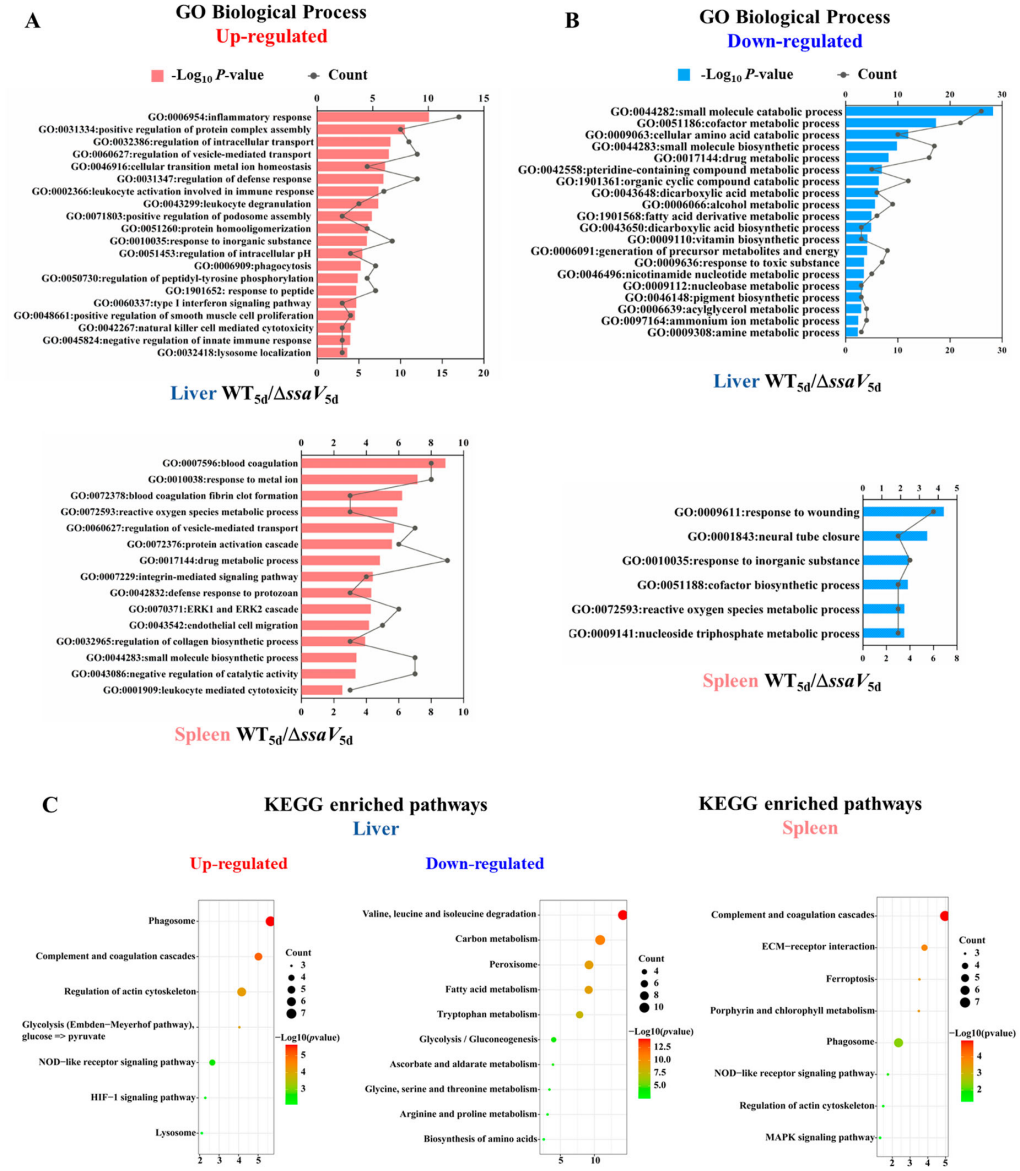
GO and KEGG analysis of the DEPs between WT and Δ*ssaV* groups: GO biological process analysis of the DEPs up-regulated (**A**) and down-regulated (**B**) in mice liver or spleen at 5 dpi. (**C**), KEGG analysis of the DEPs between WT and Δ*ssaV* in mice liver or spleen at 5 dpi.

Changes in protein levels greater than 1.5 or less than 0.67 were considered as up- or down-regulated DEPs. We then queried the Metascape database ([28]) for gene ontology (GO) annotations of these proteins in liver and spleen at 5 dpi. Figure 5(A,B) illustrate the top GO biological processes and cellular component categorization annotations. We further performed KEGG enrichment analysis of DEPs (Figure 5(C)).

As shown in Figure 5(A), proteins associated with inflammatory responses, intracellular transport, and immune regulation were higher in the WT-infected liver samples comparing to that in the MT-infected samples. Likewise, proteins involved in kinase signalling pathway, response to external stimulus such as metal ion, and immune regulation are higher in WT-infected spleens than that of the MT-infected samples. For examples, the signal transducer and activator of transcription 1 (STAT1), Chitinase-3-like protein 3 (CHIL3), Integrin Subunit Beta 2 (ITGB2), Guanylate Binding Protein 5 (GBP5), Serum Amyloid A1 (SAA1) and Serum Amyloid A2 (SAA2) were higher in WT *Salmonella* infected samples, suggesting that SPI-2 may contribute to inflammatory response during systemic infection. Higher levels of Hemopexin (Hpx), Heme Oxygenase 1 (HMOX1), Lipocalin-2 (LCN2), and Ceruloplasmin (CP) in the WT-infected samples suggests that SPI-2 is involved in iron homeostasis consistent with previous *Salmonella* intracellular growth needs.

Proteins that are lower in the liver and spleen samples infected by the WT are mainly involved in metabolic processes, including the metabolism of fatty acids, vitamins, cholesterol, and amino acid (Figure 5(B)). For example, WT *Salmonella* infection resulted in reduced expression levels of proteins associated with fatty acid β-oxidation, such as Peroxisomal acyl-coenzyme A oxidase 1 (ACOX1), Fatty Acid Binding Protein 1 (FABP1), Acetyl-CoA Acetyltransferase 1 (ACAT1), Electron Transfer Flavoprotein Subunit Beta (ETFB) and 3-ketoacyl-CoA thiolase A (Acaa1a). These data suggest that SPI-2 is involved in modulating the metabolic process during *S.* Typhimurium infection.

Next, we performed KEGG enrichment analysis of DEPs in the liver and spleen samples with a criterion of *P* < 0.01 (Figure 5(C)). Proteins associated with actin cytoskeleton, phagosome/lysosome maturation, HIF-1 signalling, and NOD-like receptor signalling were enriched in WT-infected liver samples. Similar enrichment patterns were observed from spleen samples. Interestingly, proteins associated with amino acid biosynthesis and metabolism, carbon metabolism, ascorbate and aldehyde metabolism, peroxisomes, glycolysis/glyoxalate production, fatty acid degradation were enriched in MT-infected liver samples, but not in the spleen samples. Notably, glycolysis (Embden-Meyerhof pathway) was enhanced in the DEPs of WT-infected vs. MT-infected groups. This suggests that the alterations in the glycolytic pathway may be conferred by SPI-2.

### 3.5. Protein–protein interaction analysis of differentially expressed proteins

**Figure 6.**
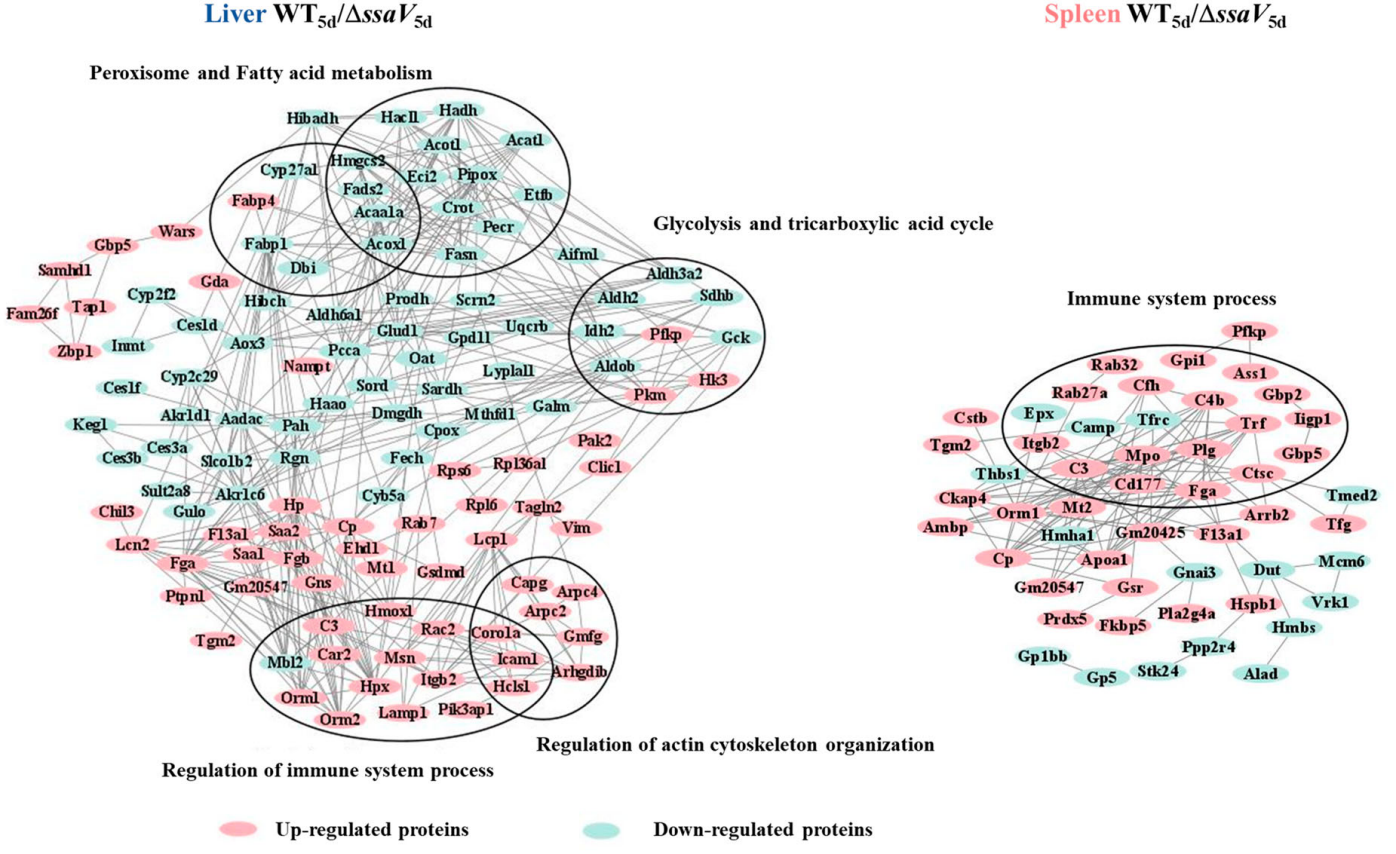
Protein-protein interaction network of DEPs identified from mice liver and spleen at 5 dpi infected with WT *S.* Typhimurium or Δ*ssaV* mutant.

To analyse potential functional roles of DEPs identified above, protein–protein interaction (PPI) analysis was performed through STRING (one of the online databases of biological protein association network) and visualized with Cytoscape 3.8.0. PPI analysis was carried out using liver and spleen DEPs from the WT-infected groups vs. Δ*ssaV* MT-infected groups at 5 dpi (Table S1–S2 in Supporting Information). DEPs with moderate confidence scores (> 0.4) were selected and nodes with network disconnections were removed (Figure 6).

In STRING, the network view summarizes the network of potential functional associations for DEPs. The network nodes are proteins, and the edges represent predicted associations. The interactomes of liver and spleen DEPs were comprised of 137 and 68 nodes. PPI analysis showed that SPI-2 induced DEPs in both liver and spleen formed correlated networks of interactions in immune responses (Figure 6(A,B)). SPI-2-induced DEPs also showed interaction networks in actin cytoskeleton organization, glycolysis and TCA cycle, peroxisome and fatty acid metabolism in mouse liver (Figure 6(A)). Results from PPI analysis are consistent with results from GO and KEGG pathway enrichment analysis described above. Thus, SPI-2 effectors may play important roles in host immune response and lipid metabolism.

### 3.6. Western blot validation

**Figure 7.**
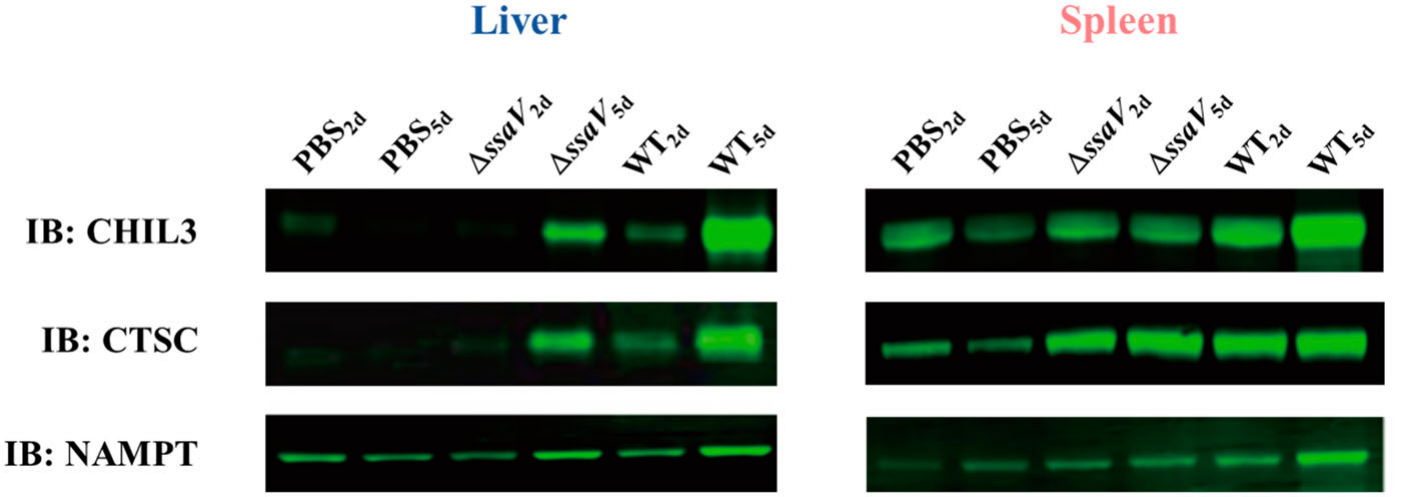
Western blot analysis of CHIL3, CTSC and NAMPT in mice liver and spleen after *S.* Typhimurium infection.

Three candidate proteins (CHIL3, CTSC and NAMPT) from the mass spectrometry data were selected to verify their expression levels by WB. Total protein levels were used for normalization in each sample group (Figure S3). Consistent with the LC-MS/MS data, expressions of Chitinase-3-like protein 3 (CHIL3), Cathepsin C (CTSC) and nicotinamide phosphoribosyltransferase (NAMPT) were significantly increased in liver and spleen samples from WT-infected mice compared to that of Δ*ssaV* MT-infected mice (Figure 7).

## 4. Discussion

*Salmonella* species are gram-negative enterobacteria that can cause diseases ranging from a self-limiting enterocolitis to systemic infection [29]. The Type III secretion system encoded by SPI-2 plays a pivotal role in intracellular survival and systemic infection. We aimed to investigate the host response triggered by SPI-2 in this study. Thus, protein levels from WT and SPI-2 defective mutant (Δ*ssaV*) infected liver and spleen samples were analysed using a quantitative proteomics approach. The phenotypes of the *ssaV* deletion strain and its corresponding complementing strain were verified using tissue culture cells based on the reported replication assay [30]. Two time points, 2 and 5 dpi, were chosen for the infection experiments because replication differences between the WT-infected and Δ*ssaV* MT-infected groups started to appear at 2 dpi, as confirmed by tissue bacterial load experiments, and 5 dpi was chosen as the endpoint because mice began to die thereafter. In our experiments, a series of DEPs were identified, and three DEPs were validated by Western blot. CHIL3, also known as Ym-1, is a marker expressed by M2 macrophages [23,31–33], and has previously been used to examine the M2 polarization of macrophages induced by *Salmonella* infection *in vitro* and *in vivo* [31,32]. CTSC is a lysosomal cysteine protease involved in M1 polarization of macrophages [34], and CTSC expression has been shown to increase in *S.* Typhimurium-infected RAW264.7 cells [23]. NAMPT exists in intracellular NAMPT (iNAMPT) and extracellular NAMPT (eNAMPT) forms. The iNAMPT is the rate-limiting enzyme in the nicotinamide adenine dinucleotide (NAD^+^) salvage pathway [35], and NAD^+^ is an essential coenzyme in aerobic glycolysis [36]. The eNAMPT is important for the differentiation of resting monocytes toward M2 macrophages [36]. *Salmonella* infection causes significant upregulation of the *NAMPT* gene in mice [33].

**Figure 8.**
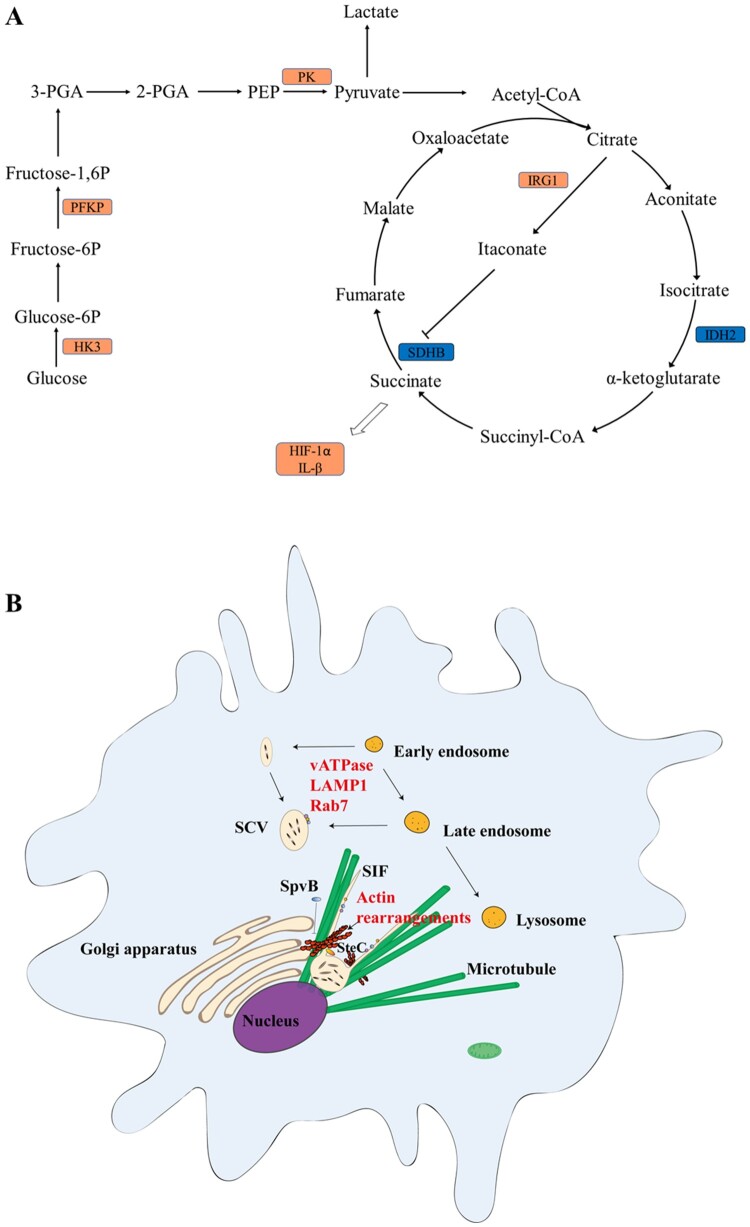
Host responses triggered by *S.* Typhimurium SPI-2 effectors during systemic infections. (**A**), SPI-2 effectors may triger host cell metabolic reprogramming and inflammation. WT-Infected cells produce ATP through aerobic glycolysis, rather than relying primarily on oxidative phosphorylation. The TCA cycle was repressed and led to increased accumulation of TCA intermediates itaconate and succinate. (**B**), Proteins associated with actin polymerization/depolymerization were up-regulated in WT-infected cells comparing to that of the Δ*ssaV* MT-infected cells.

Compared with the Δ*ssaV* MT-infected samples, liver and spleen samples from WT-infected mice exhibited significant reprogramming of cellular processes, such as glycolysis and amino acid degradation. Our results showed that host cells undergo metabolic reprogramming and activate inflammatory pathways in response to *Salmonella* infection. *Salmonella* systemic infection induces an up-regulation of glycolytic process and a repression of the TCA cycle in host cells. Specifically, hexokinase-3 (HK3), phosphofructokinase (PFKP) and pyruvate kinase (PK) were up-regulated in WT-infected samples at 5 dpi, suggesting that the glycolytic pathway was promoted because these proteins are rate-limiting enzymes involved in the glycolytic pathway. Meanwhile, isocitrate dehydrogenase (IDH2) and succinate dehydrogenase (SDHB) were down-regulated, leading to a decrease in TCA cycling and the inevitable accumulation of TCA intermediates itaconate and succinate (Figure 8(A)). Although glycolysis produces ATP less efficiently than oxidative phosphorylation, the rate of ATP production is faster. Cells may have lower oxygen levels in an infectious/inflammatory environment, so increased glycolytic metabolism may be a self-protective mechanism for cells to maintain cellular ATP levels and cell viability during a robust immune response. This is consistent with previous reports that lipopolysaccharides (LPS) stimulates a metabolic transition toward aerobic glycolysis in mouse dendritic cells [37]. Itaconate can restrict the replication of *Salmonella* in host cells during infection [38]. The accumulation of succinate may serve as an inflammatory signal by stabilizing hypoxia-inducible factor 1α (HIF-1α) and further activating IL-1β [39].

Mice liver and spleen have large numbers of macrophages, that are capable of initiating innate immune responses through phagocytosis and cytokine release. Activated macrophages may be divided into two subgroups: classical activated macrophages (M1) and alternately activated macrophages (M2). M1 macrophages perform anaerobic glycolysis to avoid diverting oxygen from NADPH oxidase activity. M1 subtype is mainly associated with inflammatory responses and can be generated by LPS and IFN-γ stimulation during infections. In contrast, M2 macrophages are mostly associated with tissue remodeling, resolution of inflammation, and anti-inflammatory responses. M2 subtype prefers oxidative phosphorylation to produce ATP. Our data suggested that WT-infected macrophages underwent M1 polarization compared to that of the Δ*ssaV* group. This reprogramming of host cells during systemic *Salmonella* infection share similarity to the metabolic changes in tumor cells, which is commonly referred to as the Warburg Effect [40]. Previous studies have shown that *S.* Typhimurium-infected macrophages promoted M2 macrophage subtype [41–44]. Our WB results showed that the level of CHIL3 was 5.3-fold higher in the WT group compared to that of the Δ*ssaV* group at 5 dpi, suggesting the existence of M2 macrophages. It is possible that *S.* Typhimurium-infected macrophages undergo both M1 and M2 polarization during different stages of the infection. The exact timing requires more detailed future studies.

Manipulation of the actin cytoskeleton occurs both when *Salmonella* invades the host cell and during its intracellular survival and replication after internalization. Our results showed proteins related to actin cytoskeleton organization were up-regulated in WT-infected samples (Figure 8(B)), such Rab7 GTPase, coronin 1A (Coro1A) and intercellular adhesion molecule-1 (ICAM-1). Rab7 plays an essential role in host endocytic trafficking [45,46]. Coro1A is known to promote F-actin disassembly [47]. Functional SPI-2 T3SS is essential for the assembly of F-actin meshwork around SCVs, whereas Δ*ssaV* MT (SPI-2 defective) shows little accumulation of F-actin around intracellular bacteria [48]. SpvB and SteC, two SPI-2 T3SS effectors, have been reported to manipulate the cytoskeleton of actin [48–51]. Our results further suggest that SPI-2 is involved in altering actin cytoskeleton to facilitate SCV biogenesis and perhaps even the release of bacteria later in the infection process.

Overall, we aimed to use a differential proteomics approach to analyse the alterations in host protein levels triggered by SPI-2 during systemic *Salmonella* infection. On the one hand, our experiments capture changes from some of the reported host proteins triggered by SPI-2 effectors. This both validates the reliability of our data and provides additional information and evidence against some proteins whose functions are currently controversial. For example, our results showed that the expression of LCN2 and HMOX1 was significantly enhanced in the WT-infected group compared with the MT-infected group at 5 dpi. LCN2 interferes with bacterial access to siderophore-bound iron, and enhances the production of pro-inflammatory cytokines [52], while iron ion efflux increases SPI-2 protein expression [53,54]. Our results are consistent with the above reports. The role of HMOX1 in protecting against *Salmonella* infection remains controversial due to conflicting findings [55], and our results may provide more information to further reveal the function of HMOX1. On the other hand, we identified a number of new host proteins potentially triggered by SPI-2. Some of them, such as tyrosine-protein phosphatase non-receptor type 1 (PTPN1), serum amyloid A-1 protein (SAA1) and chloride intracellular channel protein 1 (CLIC1), although previously reported in *Salmonella* infections, we have for the first time clearly associated them with SPI-2 effectors. We also identified some new host proteins that have never been reported in *Salmonella* infection previously, such as indolethylamine *N*-methyltransferase (INMT) and peroxisomal trans-2-enoyl-CoA reductase (PECR). The predicted model was described in Figure 8 based on our proteomics data, and the model needs to be further tested experimentally. All these results provide important information to reveal the role of SPI-2 in the process of systemic *Salmonella* infection and will further guide the study of the molecular mechanism of SPI-2 in the future.

## 5. Conclusion

In summary, we used a differential proteomic approach to analyse host proteins levels in mice liver and spleen during systemic *S.* Typhimurium infection. Our results show that systemic *S.* Typhimurium infection induces up-regulation of glycolytic processes and a repression of the TCA cycle, triggering metabolic reprogramming and inflammatory responses in host cells. WT-infected tissues prefer to generate energy through aerobic glycolysis rather than relying on oxidative phosphorylation. Also, infected macrophages may undergo both M1 and M2 polarization. In addition, our results further suggest that SPI-2 is involved in altering actin cytoskeleton to facilitate the SCV biogenesis. Although the model predicted by our proteomics data needs further experimental validation in the future, our results provide valuable insights into the role of SPI-2 during systemic *Salmonella* infection and will guide future studies to dissect the molecular mechanisms of how SPI-2 functions *in vivo*.

## Supplementary Material

Supplemental Material
