# Supplementary material for: Host metabolic shift during systemic Salmonella infection revealed by comparative proteomics
Source: Emerg Microbes Infect. 2021 Sep 17;10(1):1849–61. doi: 10.1080/22221751.2021.1974316 (PMC8451668; doi:10.1080/22221751.2021.1974316)
Supplement: Supplemental Material [file TEMI_A_1974316_SM1439.docx]

Supporting Information

**Host Metabolic Shift During Systemic *Salmonella* Infection Revealed by Comparative Proteomics**

Yuanyuan Wang^1^, Chunmei Wu^2^, Jiacong Gao^1^, Xudong Du^1^, Xiangyun Chen^1^, Haimin Lei^2^, Mei Zhang^2^*

**^1^** TEDA School of Biological Sciences and Biotechnology, Nankai University, Tianjin 300457, China;

**^2^** School of Chinese Materia Medica, Beijing University of Chinese Medicine, Beijing 102488, China.

*Corresponding Author:

Prof. Mei Zhang

[meizhang@bucm.edu.cn](mailto:meizhang@bucm.edu.cn)

**Contents**

1. **Materials and methods**
   1. **Bacterial strains and animal experiments**
   2. **Analysis of *S.* Typhimurium loads in tissues**
   3. **Histopathology**
   4. **Protein sample preparation**
   5. **High-pH reversed-phase peptide fractionation**
   6. **Nanoflow LC-MS/MS analysis**
   7. **Proteomic data processing and bioinformatics analysis**
   8. **Western blot**
2. **Results in supporting information**
   1. **Histopathology**
   2. **Reproducibility of experiments**
   3. **Western blot validation**
3. **The Differentially expressed proteins between the WT- and** Δ***ssaV* MT-infected groups**
4. **References in Supporting Information**

**1. Materials and methods**

**1.1 Bacterial strains and animal experiments**

*S.* Typhimurium wild-type (WT) strain SL1344 [1] and its *∆ssaV* mutant (MT) were used in this study. *S.* Typhimurium strains were routinely cultured in Luria-Bertani (LB) broth, and 25 μg/mL streptomycin was used when applicable. The mouse systemic infection model used in this study was basically followed the reported approach [2]. All experimental procedures were approved by the Animal Care and Use Ethics Committee of Nankai University (License No. NKU20160816). Briefly, female C57BL/6 mice (6- to 8-week old, obtained from the Beijing Vital River Laboratory, Beijing, China) were randomly divided into differnet groups (6 mice for each mixture sample) and housed in independently ventilated cages under controlled conditions. Water and food were withdrawn 4 h before per os (p.o.) treatment with 20 mg of streptomycin. Afterward, mice were supplied with water and food ad libitum. At 20 h after streptomycin treatment, water and food were withdrawn again for 4 h before the mice were infected with 10^8^ CFU of *S.* Typhimurium (100 µL suspension in PBS p.o.) or treated with sterile PBS. Thereafter, drinking water ad libitum was offered immediately and food 2 h postinfection. Mice were anesthetized under ether narcotization at the time points of 2 days postinfection (dpi) and 5 dpi, respectively. Before sacrificing, cardiac perfusion was carried out to drain the remaining blood in tissues. Three biological replicates (BR) were performed for each condition.

**1.2 Analysis of *S.* Typhimurium loads in tissues**

To analyze the colonization, livers, spleen, and mesenteric lymph nodes were aseptically removed, washed by cold PBS, and homogenized in 4 °C cold PBS (0.5% Tergitol, 0.5% bovine serum albumin) by using a Potter homogenizer after being cutted into small pieces. Intestinal contents from cecum were collected, weighed and then resuspended in 500 μL of 4 °C PBS at the indicated postinfection time points. Appropriate dilutions were carried out and plated onto Mac-Conkey agar plus streptomycin (50 μg/mL) plates for output enumeration. The minimal detectable value was 10 CFU/organ.

**1.3 Histopathology**

Tissue segments of the liver, spleen, mesenteric lymph nodes, and cecum were fixed and embedded in paraffin according to standard procedures, including fixed in 10% Neutral Buffered Formalin (NBF), dehydrated in a series of graded ethanols, and embedded in paraffin. And then, samples were sectioned and stained with hematoxylin and eosin (H&E).

**1.4 Protein sample preparation**

The comparative proteomic based on stable isotope dimethyl labeling technique is shown in Figure 1 in the main text. Each tissue sample was ground in liquid nitrogen and lysed in lysis buffer containing 100 mM NH_4_HCO_3_ (pH 7.8), 8 M urea, 4% CHAPS, 65 mM dithiothreitol (DTT) and protease inhibitors (Complete mini protease inhibitor cocktail, Roche, Basel, Switzerland), following a 2 min sonication on ice (2 sec ON/2 sec OFF pulses at 42% amplitude). Next, each sample was centrifuged at 10,000 *g* for 30 min at 4 ℃ (Eppendorf, Hamburg, Germany), and the supernatant was transferred into a new tube. We added 4 volumes of cold acetone to each supernatant, mix well, and placed it at -20 °C for 4 h to precipitate proteins. Pellets were centrifuged, dried, and dissolved in triethylammonium bicarbonate (TEAB) buffer containing 8 M urea.

Two hundred micrograms of proteins were reduced by 50 mM DTT at 37 °C for 1 h. Alkylation was performed in a 100 mM iodoacetamide (IAA) solution at room temperature for 1 h in the dark. After alkylation, the sample was diluted using a 100 mM TEAB buffer to give a final urea concentration of 1 M. Tryptic digestion was then performed at a protein-to-trypsin concentration ratio of 25:1 (w/w) for 20 h at 37 °C.

Stable isotope dimethyl-labeling of each tryptic digested peptide sample (50 µg peptide/sample) was carried out according to the reported protocol [3]. The concentrations of peptide samples were determined using the commercial kit (Pierce™ Quantitative Colorimetric Peptide Assay, ThermoFisher, USA). In our research, PBS-treated groups were “light labeled” with the combination of regular formaldehyde (CH_2_O) and sodium cyanoborohydride (NaBH_3_CN). WT-infected groups were “intermediate labeled” with the combination of deuterated formaldehyde (CD_2_O) and NaBH_3_CN. The ∆*ssaV* MT-infected groups were “heavy labeled” with the combination of ^13^C-labeled deuterated formaldehyde (^13^CD_2_O) and sodium cyanoborodeuteride (NaBD_3_CN). In reverse label experiment, the PBS group and ∆*ssaV* group reagents were changed to each other, whereas the SL1344 group was kept constant. When analyzing samples at different time points, the light, intermedium and heavy labeled samples were mixed at 1:1:1 ratio. Samples were desalted using a C_18_ SPE column (SEP-PAK C18, Waters, MA, USA) and dried by vacuum centrifugation for next step.

**1.5 High pH Reversed-Phase Peptide Fractionation**

All samples were fractionated on a Shimadzu LC-20AT HPLC system equipped with an High-pH-Reverse-phase column (XBridge BEH130 C18 column, 130 Å, 5 μm, 4.6 mm × 250 mm) at a flow rate of 0.5 mL/min. Each desalted peptide sample (100 μg) was dissolved in 40 μL mixture solution of 95% buffer A and 5% buffer B, v/v. The mobile phase consisted of buffer A (98% H_2_O, pH 10) and buffer B (98% acetonitrile, pH 10). The gradient elution for buffer B was as follows: 5% from 0 to 4 min, 5−8% from 4 to 6 min, 8−24% from 6 to 40 min, 24−34% from 40 to 58 min, 34−80% from 58 to 60 min, 80% from 60 to 64 min, 80−5% from 64 to 80 min. The collected 36 fractions were combined into 12 samples and lyophilized.

**1.6 Nanoflow LC-MS/MS analysis**

Experiments were performed on an hybrid quadrupole-orbitrap Q-Exactive® mass spectrometer coupled to a nanoflow Liquid Chromatography EASY-nLC 1200 (Thermo Fisher Scientific, San Jose, CA, USA) equipped with a trap column (3 μm particle, 75 μm × 2 cm, 100 Å) and an analytical column (2 μm particle, 75 μm × 25 cm, 100 Å) at a fixed flow rate of 260 nL/min. Samples were dissolved in 20 μL H_2_O with 0.1 % FA for LC-MS/MS analysis. The mobile phase consisted of buffer A (0.1 % FA in H_2_O) and buffer B (0.1 % FA in ACN). The gradient elution for buffer B was as follows: 3−8% from 0 to 3 min, 8−35% from 3 to 75 min, 35−100% from 75 to 84 min, 100% from 84 to 90 min. MS1 spectra were operated in data-dependent acquisition (DDA) full scan positive mode, scanning 300 to 1800 *m/z* with a resolution of 70,000. The top 10 most intense ions from each MS1 scan were selected for collision‐induced dissociation (CID).

**1.7 Proteomic data processing and bioinformatics analysis**

Raw files were processed by Proteome Discoverer 2.2 and searched against the proteome database of C57/BL6 downloaded from Uniprot (<http://www.uniprot.org/>). A maximum of two missed cleavages were allowed for tryptic digestion. The carbamidomethylation (+57.0215 Da) on cysteine, and dimethylation (light labeled: +28.031 Da; intermediate labeled: +32.056 Da; heavy labeled: +36.076 Da) on lysine and *N*-terminal amine were set as static modifications. The oxidation (+15.995 Da) and acetylation (+42.011 Da) on *N*-terminal amine were set as dynamic modifications. The protein and peptides less than 1.0% false discovery rate (FDR) were accepted. Only proteins with at least two unique peptides were considered as qualitable. Two or more identified quantification ratios (including reverse-labeled) were used to quantify. We used “Wu Kong” platform (<https://www.omicsolution.org/wkomics/main/>) for replacing miss values and hypothesis testing. Missing values were replaced by the SeqKnn method. The *P*-values were calculated via Student’s t-test and further corrected with multiple hypothesis testing using the Banjamini-Hochberg procedure. Proteins with an average fold-change > 1.5 or < 0.67 with *P* < 0.05 were considered as differentially expressed proteins (DEPs). The metascape database (https://metascape.org) was used to perform Gene Ontology (GO) annotation [4] and KEGG pathway analysis [5].

**1.8** **Western** **blot**

SDS-PAGE (12.5% gel) was performed with a Bio-Rad Mini Protean III apparatus followed electroblotting on 0.45 µm PVDF membrane (GE). After transfer, PVDF membranes were stained by LI-COR REVERTTM [6]. Briefly, membranes were incubated in total protein stain for 5 minutes, and imaged in the 700 nm channel of the Odyssey imaging system (LI-COR). After being blocked for 1 h at room temperature in 5% NFDM/TBST (BD), membranes were incubated at 4 ℃ overnight with primary antibodies: anti-Chil3 antibody (Abcam,1:50000), anti-Nampt (Abcam, 1:1000) and anti-Ctsc (Santa Cruz, 1:1000). And then, membranes were washed with TBST buffer and incubated in TBST containing secondary antibodies for 1 h at room temperature: IRDye 800CW Goat anti-Rabbit IgG (LI-COR, 1:15000) and IRDye 800CW Goat anti-Mouse IgG (LI-COR, 1:15000). Finally, membranes were analyzed by Odyssey CLX in the 800 nm channel.

**2. Results in supporting information**

**2.1 Histopathology**

Since the early stage (2 dpi) of systemic infection, the cecum of both *S.* Typhimurium Δ*ssaV* MT- and WT-infected mice (Figure S1A and S1B) showed disruption of the epithelial layer and mild infiltration of inflammatory cells within the lamina propria compared with the control group (Figure S1E). At 5 dpi, cecum sections Δ*ssaV* MT-infected mice showed marked mucosal disruption, degeneration of the epithelial lining of the crypts and infiltration of inflammatory cells within the lamina propria (Figure S1C). Also, these phenomena were more severe in the WT-infected group at the same systemic infection stage (Figure S1D). However, in that it appears that mesenteric lymph node samples from WT- and Δ*ssaV* MT-infected mice were not shown to be significantly different, either in early or late stages of systemic infection (Figure S1F-S1J).

**
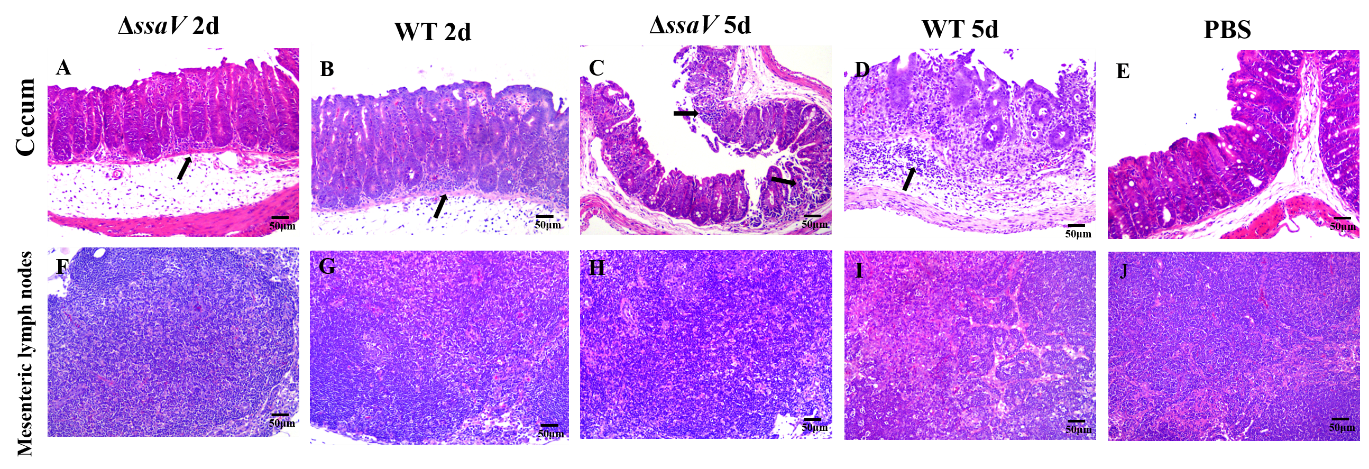
**

**Figure S1.** Hematoxylin-Eosin (H&E) stained histopathological sections of cecum and mesenteric lymph node of WT *S.* Typhimurium or Δ*ssaV* mutant-infected mice at early (2 dpi) and late (5 dpi) stages of systemic infection (HE, ×100; Bar: 50 μm). Black arrows indicated infiltration of inflammatory cells.

**2.2 Reproducibility of experiments**

We investigated the reproducibility of our experiments. Pairwise comparisons of the coefficients of variation (CV) between different groups have been plotted (Figure S2), indicating the good biological reproducibility of our experiments.


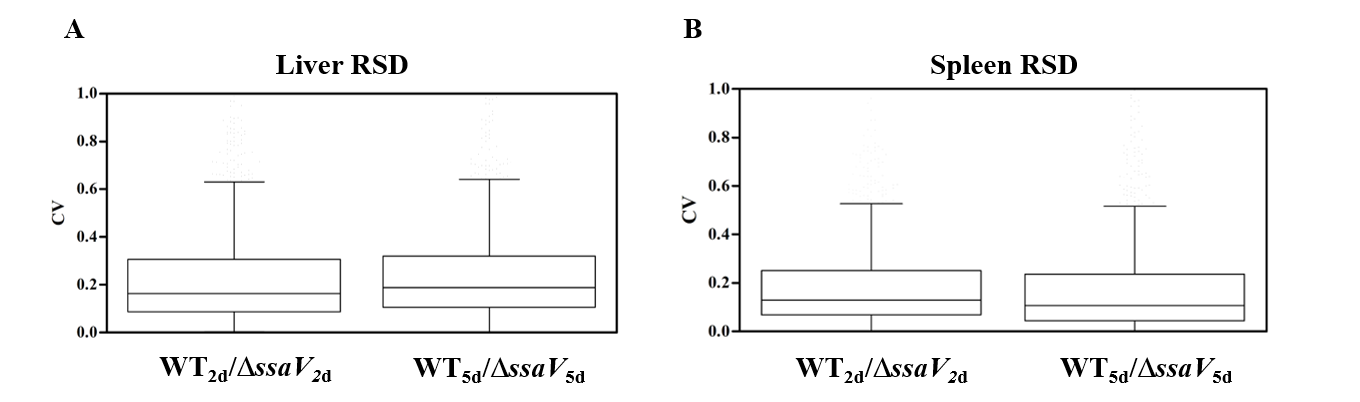


**Figure S2.** Box plots visualizing coefficients of variations (CVs) of identified mice hepatic and splenic proteins at 2 dpi and 5 dpi. Box plots showed with 25^th^ percentile, median and 75^th^ percentile values.

**2.3 Western blot validation**

Expression changes of three candidate proteins (CHIL3, CTSC and NAMPT) from the mass spectrometry data were verified by Western blot (WB). Total protein staining of each sample was used for normalization (Figure S3).


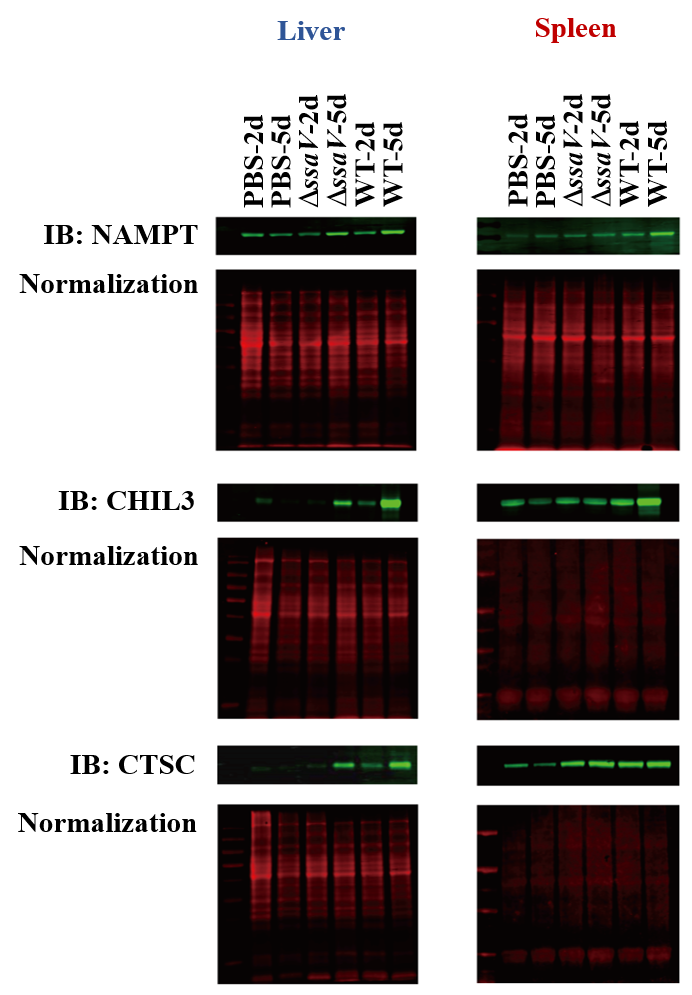


**Figure S3.** Western blot analysis of CHIL3, CTSC and NAMPT in mice liver and spleen after *S.* Typhimurium infection. Total protein staining of each sample was used for normalization.

**3. The Differentially expressed proteins between the WT- and Δ*ssaV* MT-infected groups**

The DEPs between WT- and Δ*ssaV* MT-infected groups were summarized in Table S1. Table S1 will be provided as a separate appendix.

**4. References in Supporting Information**

1. Hoiseth SK, Stocker BA. Aromatic-dependent Salmonella typhimurium are non-virulent and effective as live vaccines. Nature. 1981;291(5812):238−239. doi: 10.1038/291238a0.
2. Barthel M, Hapfelmeier S, Quintanilla-Martínez L, et al. Pretreatment of mice with streptomycin provides a Salmonella enterica serovar Typhimurium colitis model that allows analysis of both pathogen and host. Infect Immun. 2003;71(5):2839−2858. doi: 10.1128/IAI.71.5.2839-2858.2003.
3. Boersema PJ, Raijmakers R, Lemeer S, et al. Multiplex peptide stable isotope dimethyl labeling for quantitative proteomics. Nat Protoc. 2009;4(4):484−494. doi: 10.1038/nprot.2009.21.
4. Ashburner M, Ball CA, Blake JA, et al. Gene ontology: tool for the unification of biology. The Gene Ontology Consortium. Nat Genet. 2000;25(1):25–29. doi: 10.1038/75556.
5. Kanehisa M, Goto S. KEGG: kyoto encyclopedia of genes and genomes. Nucleic Acids Res. 2000;28(1):27–30. doi: 10.1093/nar/28.1.27.
6. Kirshner ZZ, Gibbs RB. Use of the REVERT^®^ total protein stain as a loading control demonstrates significant benefits over the use of housekeeping proteins when analyzing brain homogenates by Western blot: An analysis of samples representing different gonadal hormone states. Mol Cell Endocrinol. 2018;473:156–165. doi: 10.1016/j.mce.2018.01.015.
